# Adaptogenic effect of *Morus alba* on chronic footshock-induced stress in rats

**DOI:** 10.4103/0253-7613.59921

**Published:** 2009-12

**Authors:** Vandana S. Nade, Laxman A. Kawale, Rashmi A. Naik, Adhikrao V. Yadav

**Affiliations:** Department of Pharmacology, M.V.P. Samaj's College of Pharmacy, Gangapur Road, Nashik - 422002, India; 1 Goa College of Pharmacy, Panaji - 403 001, India

**Keywords:** Adrenal gland, antistress, footshock stress, memory, *Morus alba*

## Abstract

**Objective:**

The objective of the present study was to evaluate the adaptogenic property of the ethyl acetate-soluble fraction of methanol extract of *Morus alba* roots against a rat model of chronic stress (CS).

**Materials and Methods:**

Rats were exposed to stress procedure for 21 days. The stress procedure was mild, unpredictable footshock, administered for 1 h once daily for 21 days. Rats were administered with the ethyl acetate soluble fraction of methanol extract of *M. alba* roots (25, 50 and 100 mg/kg p.o) 1 h before footshock for 21 days and behavioral parameters were evaluated for cognitive dysfunction and depression using elevated plus maze and despair swim test, respectively. On day 21, rats were sacrificed immediately after stress and blood was collected for biochemical estimation. The adrenal gland and spleen were dissected for organ weight and the stomach was dissected for ulcer score.

**Results:**

CS significantly induced cognitive deficit, mental depression and hyperglycemia and increased blood corticosterone levels, gastric ulcerations and adrenal gland weight, but decreased the splenic weight. Pre-treatments with the ethyl acetate soluble fraction of methanol extract of *M. alba* roots (25, 50 and 100 mg/kg, p.o.) significantly attenuated the CS-induced perturbations. Diazepam (1 mg/kg, p.o.) was used as the standard antistress drug.

**Conclusion:**

The results indicate that *M. alba* possesses significant adaptogenic activity, indicating its possible clinical utility as an antistress agent.

## Introduction

Adaptogens are the plant-derived biologically active substances that appear to induce a state of non-specific increase of resistance of the organism to diverse assaults that threaten internal homeostasis and improve physical endurance.[1] Supplementation with various macro and micronutrients and herbal preparations has been evaluated for their adaptogenic activity during exposure to a stressful environment.[2]

*Morus alba* (Moraceae), commonly known as White mulberry, has been used since ancient times in folk medicine for its many medicinal properties. It is cultivated extensively throughout India. The plant has been used in Indian folklore as a nervine tonic.[3] This widely grown plant has been in use by tribals of this country for ailments such as asthma, cough, bronchitis, edema, insomnia, wound healing, diabetes, influenza, eye infections and nosebleeds.[4] It has been extensively studied for its antidiabetic,[5] antibacterial,[6] antianaphylactic,[7] neuroprotective,[8] hypolipidemic,[9] hypotensive[10] and anti-anxiety activities.[11] A number of earlier investigations indicated that *M. alba* exhibits an activity profile that may reflect a putative antioxidative stress effect. The antioxidant potency of some phenolic compounds from *M. alba* has been reported.[12] The plant is a very good source of ascorbic acid, of which over 90% is present in a reduced form, and also contains carotene, Vitamin B1, folic acid, folinic acid, isoquercetin, quercetin, tannins, flavonoids and saponins, which act as a good source of natural antioxidants.[4]

A large number of medicinal plants have been reported to possess adaptogenic activity. However, there is no report available on the adaptogenic activity of *M. alba*. Hence, the present work was undertaken to investigate the antistress adaptogen potential of the ethyl acetate soluble fraction of *M. alba* in chronic footshock stress using biochemical and behavioral approach.

## Materials and Methods

### Plant material

The roots of *M. alba* were collected in the month of October 2007 from a local area in Nashik, India, and authenticated by P. S. N. Rao (Director, Botanical Survey of India, Pune). A voucher specimen of the plant has been deposited at the Botanical Survey of India, Pune (Voucher Specimen No. NVMA2). The roots were shade-dried and reduced to a coarse powder. The powdered plant material was defatted using petroleum ether (60–80°C) using a Soxhlet extractor and then extracted with methanol for 72 h to obtain the extract. The extract was filtered and concentrated under reduced pressure. The yield of methanol extract of *M. alba* roots was found to be 18.25% w/w. The methanolic extract was exhaustively extracted with ethyl acetate to obtain ethyl acetate soluble (EASF 10.3% w/w) and ethyl acetate insoluble fractions (EAIF 6.1% w/w). Suspensions of EASF was prepared in distilled water using Tween 80 (0.2% v/v) as a suspending agent. The extract was administered in doses of 25, 50 and 100 mg/kg per orally (p.o.) 60 min before the footshock. Control group was given only vehicle (0.2% v/v, Tween 80) in volume equivalent to that of the plant extracts.

### Animals

Male Wistar rats (150–180 g) were used for the study. The animals were housed in polypropylene cages and maintained at 25° ± 2°C, 12:12-h light:dark cycle and 50 ± 5% RH with free access to food and water ad libitum. Animals were acclimatized to laboratory conditions before the test. All the experiments were carried out during the light period (08:00–16:00 h). The studies were carried out in accordance with the guidelines given by the Committee for the Purpose of Control and Supervision of Experiments on Animals (CPCSEA), New Delhi (India). The Institutional Animal Ethical Committee of M.V.P.S. College of Pharmacy, Nashik, approved the protocol of the study (IAEC/2008/01).

### Drugs and chemicals

Diazepam injection I.P. (Ranbaxy Laboratory Ltd., Gurgaon, India) was used as the standard drug. GOD-POD (Glucose Oxidase-Peroxidase) Kit (Pathozyme Diagnostics, Kolhapur, India) was used for biochemical investigations.

### Phytochemical screening of M. alba roots

Screening for the presence of phenolic compounds, tannins, flavonoids, alkaloids, glycosides, triterpenes, sterols, anthocyanins and anthroquinones was carried out using methods previously described.[13,14]

### Acute toxicity test

Acute toxicity test was performed according to the Organization for Economic Cooperation Development (OECD) guidelines No. 425. The criteria for the selection of species, doses and number of animals were according to the OECD guidelines. The EASF of *M. alba* was administered orally in doses of 100, 200, 400, 800, 1000 and 2000 mg/kg to the group of mice (n = 3) and the percentage mortality was recorded for a 24-h period. During the first 1 h after the drug administration, the mice were observed for gross behavioral changes as described by Irwin *et al.* (1968). The parameters observed were hyperactivity, grooming, convulsions, sedation, loss of righting reflex, respiration, salivation, urination and defecation.[15]

### Treatment schedule

Animals were divided into six groups, having six animals in each group. Group I: control non-stress (0.2% v/v, Tween 80); Group II: vehicle + chronic stress (CS); Group III: EASF (25 mg/kg, p.o.) + CS; Group IV: EASF (50 mg/kg, p.o.) + CS; Group V: EASF (100 mg/kg, p.o.) + CS; Group VI: diazepam (1 mg/kg, p.o.). EASF (25, 50 and 100 mg/kg) and diazepam were administered per orally to each animal daily 60 min before footshock.

### Experimental methods

Induction of chronic footshock stress

The rats were subjected to unpredictable footshock (2 mA) through a grid floor once daily for 1 h for 21 days. The duration of each shock and the interval between the shocks were randomly programmed between 3–5 and 10–100 s, respectively.[16]

### Effect of EASF of M. alba on cognitive deficits induced by chronic footshock stress in elevated plus maze

An elevated plus maze consisted of two open arms (35 cm × 5 cm) crossed with two closed arms (35 cm × 5 cm × 20 cm), which were connected together with a central square of (5 cm × 5 cm). The apparatus was elevated to a height of 50 cm in a dimly illuminated room. Rats were placed individually at the end of either of the open arms facing away from the central platform. The time taken by each animal to move from the open arm to either of the closed arms was recorded. This duration of time was called transfer latency (TL). If the animal did not enter into any of the enclosed arms within 120 s, it was gently pushed into any of the enclosed arms and TL was considered as 120 s. Each rat was explored for 5 min on the maze on day 0. TL was noted on days 1, 7, 14 and 21 after induction of stress in order to assess the retention of learning.[17]

### Effect of EASF of M. alba on behavioral depression induced by CS in the despair swim test

Rats were individually forced to swim in an open glass chamber (25 cm × 12 cm × 25 cm) containing fresh water to a height of 15 cm maintained at 25° ± 2°C. The duration of immobility was recorded for the total 5-min test period. Rats were considered to be immobile when cessation of swimming occurred, with the head floating just above the water level. Duration of immobility was noted on days 1, 7, 14 and 21.[18]

### Biochemical investigations

The rats were anesthetized under light ether. Blood was removed from the retroorbital plexus using a capillary in microsample tubes and the serum was separated and used for biochemical investigations on days 1, 7, 14 and 21.

### Estimation of blood serum glucose level

Serum glucose levels were determined using a standard Glucose Oxidase-Peroxidase kit.

### Estimation of blood serum corticosterone level

The quantitative measurement of serum corticosterone in the brain was performed by the method of Bartos *et al.*, 1979.[19]

### Gastric ulceration

On day 21, the rats were sacrificed by cervical dislocation. The stomachs were removed, opened along the greater curvature and were pinned on a cork plate. The number of ulcers and its severity was registered with a stereomicroscope. The severity of ulcer was scored as 0 = no ulcer, 1 = superficial ulcers, 2 = deep ulcers, 3 = perforations. The ulcer index (UI) was calculated.[15]

### Adrenal gland and spleen weights

After sacrificing rats, the adrenal gland and spleen were removed and weighed.[20]

### Statistical analysis

Results are expressed as mean ± SEM, and the statistical analysis of data was performed using one-way analysis of variance (ANOVA) followed by Dunnett's test. A probability level <0.05 was considered statistically significant.

## Results

### Phytochemical screening

The phytochemical screening of EASF of *M. alba* revealed the presence of phenolic compounds, flavonoids and alkaloids.

### Acute toxicity

Oral administration of EASF of *M. alba* up to 2 g/kg did not produce any toxic effects on the normal behavior of the mice. No mortality was observed and the extract was found to be safe at the given doses.

### Effect of EASF of M. alba on cognitive deficits induced by chronic footshock stress in elevated plus maze

CS significantly increased transfer latencies (*P*<0.01) as compared with the control group on days 7, 14 and 21, indicating impairment in learning and memory. Administration of EASF of *M. alba* (25, 50 and 100 mg/kg p.o.) for 21 days significantly decreased transfer latencies on days 7, 14 and 21, respectively, as compared to the CS group (*P*<0.01), whereas diazepam (1 mg/kg) did not show any significant improvement in memory [Table 1].

**Table 1:** Effect of EASF of *M. alba* on transfer latencies (s) in chronic footshock stress-induced memory deficit

*Treatment groups (mg/kg, p.o.)*	*Transfer latencies (s)*				
	
	*Day 0*	*Day 1*	*Day 7*	*Day 14*	*Day 21*
Control	67.5 ± 2.5	65.5 ± 3.6	40.0 ± 5.1	41.1 ± 2.0	42.5 ± 4.1
Vehicle	58.1 ± 1.8	59.5 ± 1.8	67.1 ± 1.5**^a^	73.3 ± 1.9**^a^	78.0 ± 2.9**^a^
Diazepam (1)	59.1 ± 1.1	57.3 ± 2.2	70.3 ± 3.3	65.8 ± 2.5	61.8 ± 2.0
EASF (25)	72.3 ± 6.7	38.0 ± 2.5*^b^	24.3 ± 3.8**^b^	21.3 ± 2.0**^b^	18.6 ± 1.7**^b^
EASF (50)	67.0 ± 5.3	31.3 ± 4.7*^b^	25.3 ± 4.7**^b^	18.3 ± 2.3**^b^	15.8 ± 1.6**^b^
EASF (100)	66.8 ± 4.1	31.0 ± 4.4*^b^	37.5 ± 4.6**^b^	43.0 ± 5.2**^b^	41.8 ± 9.1**^b^

Values are expressed as mean ± S.E.M. (n = 6)

a Compared with control treated group

b Compared with chronic stress group

* *P* < 0.05,

** *P* < 0.01. (One-way ANOVA followed by Dunnett's test).

### Effect of EASF of M. alba on behavioral depression induced by CS in the despair swim test

The duration of immobility was significantly increased in (*P*<0.01) the CS group on all days in the stress-induced behavioral despair test. Administration of EASF of *M. alba* (25, 50 and 100 mg/kg) for 21 days significantly (*P*<0.01) decreased the duration of immobility. This increased immobility was also significantly (*P*<0.01) reduced by diazepam (1 mg/kg) [Table 2].

**Table 2:** Effect of EASF of *M. alba* on depressive behavior in chronic footshock stress

*Treatment groups (mg/kg, p.o.)*	*Duration of immobility (s)*			
	
	*Day 1*	*Day 7*	*Day 14*	*Day 21*
Control	51.1 ± 2.4	47.6 ± 5.5	41.6 ± 7.5	51.1 ± 3.0
Vehicle	70.3 ± 0.7**^a^	82.1 ± 3.9**^a^	89.6 ± 5.1**^a^	93.8 ± 3.2**^a^
Diazepam (1)	59.8 ± 3.8	72.6 ± 2.8	52.8 ± 2.8**^b^	49.3 ± 4.2**^b^
EASF (25)	55.1 ± 4.1**^b^	45.1 ± 1.4**^b^	40.6 ± 0.9**^b^	38.6 ± 1.2**^b^
EASF (50)	54.8 ± 3.5**^b^	40.6 ± 3.7**^b^	43.0 ± 1.5**^b^	41.6 ± 1.4**^b^
EASF (100)	50.8 ± 2.4**^b^	36.5 ± 9.1**^b^	42.6 ± 9.7**^b^	42.1 ± 6.8**^b^

Values are expressed as mean ± SEM (*n* = 6),

a Compared with control treated group,

b Compared with chronic stress group

** *P* < 0.01 (One-way ANOVA followed by Dunnett's test.)

### Effect of EASF of M. alba on CS-induced alterations in serum glucose level

CS exposure resulted in a significant increase in the serum level of glucose (*P*<0.01). Pre-treatment with EASF of *M. alba* (25, 50 and 100 mg/kg) for 21 days significantly (*P*<0.01) decreased the serum glucose level on all days of the test as compared with the CS group. The serum glucose level was also significantly decreased in the diazepam (1 mg/kg)-treated group (*P*<0.01) [Table 3].

**Table 3:** Effect of EASF of *M. alba* on transfer latencies (s) in chronic footshock stress-induced memory deficit

*Treatment groups (mg/kg, p.o.)*	*Blood glucose level (mg%)*				
	
	*Day 0*	*Day 1*	*Day 7*	*Day 14*	*Day 21*
Control	93.0 ± 1.1	92.3 ± 1.0	95.3 ± 1.6	93.0 ± 0.9	90.6 ± 1.1
Vehicle	93.6 ± 1.5	97.3 ± 1.5*^a^	122.1 ± 1.8**^a^	130.2 ± 0.8**^a^	139.7 ± 1.9**^a^
Diazepam (1)	86.8 ± 1.7**^b^	85.8 ± 1.6**^b^	88.9 ± 1.5**^b^	87.6 ± 1.9**^b^	84.7 ± 1.9**^b^
EASF (25)	88.7 ± 1.7	90.5 ± 0.9**^b^	110.9 ± 1.9**^b^	115.2 ± 2.5**^b^	116.9 ± 2.9**^b^
EASF (50)	86.0 ± 1.1**^b^	87.6 ± 1.1**^b^	94.6 ± 1.7**^b^	92.1 ± 1.3**^b^	90.1± 1.1**^b^
EASF (100)	81.0 ± 1.3**^b^	84.2 ± 0.8**^b^	90.7 ± 1.1**^b^	88.9 ± 0.4**^b^	86.1± 0.2**^b^

Effect of EASF of *M. alba* on chronic stress-induced increase in serum glucose levels.

### Effect of EASF of M. alba on CS-induced alterations in serum corticosterone level

Exposure to CS significantly increased the serum corticosterone level (*P*<0.01). Pre-treatment with EASF of *M. alba* (25, 50 and 100 mg/kg) significantly (*P*<0.01) decreased the corticosterone level as compared to the CS group. The serum corticosterone level was also significantly decreased in the diazepam (1 mg/kg)-treated group (*P*<0.01) [Table 4].

**Table 4:** Effect of EASF of *M. alba* on chronic stress-induced increase in serum corticosterone levels

*Treatment groups (mg/kg, p.o.)*	*Serum corticosterone level (μg/dl)*				
	
	*Day 0*	*Day 1*	*Day 7*	*Day 14*	*Day 21*
Control	16.4 ± 1.7	16.9 ± 1.4	15.4 ± 1.3	14.4 ± 1.4	12.1 ± 1.1
Vehicle	13.3 ± 1.9	12.8 ± 2.4	19.1 ± 2.2	24.4 ± 2.8**^a^	27.5 ± 3.5
Diazepam (1)	13.4 ± 1.2	14.3 ± 1.3	17.0 ± 1.3	16.4 ± 1.4*^b^	15.9 ± 1.3**^b^
EASF (25)	16.2 ± 1.6	14.1 ± 0.8	16.3 ± 1.5	17.7 ± 1.4*^b^	18.5± 1.6**^b^
EASF (50)	16.4 ± 1.7	16.0 ± 2.2	18.2 ± 1.4	17.0 ± 1.8*^b^	16.3± 1.5**^b^
EASF (100)	14.9 ± 1.9	13.0 ± 1.1	15.8 ± 1.2	14.9 ± 0.8**^b^	13.2± 0.4**^b^

Values are expressed as mean ± SEM (*n* = 6),

a Compared with control treated group,

b Compared with chronic stress group

* *P* < 0.05,

** *P* < 0.01 (One-way ANOVA followed by Dunnett's test.)

### Effect of EASF of M. alba on CS-induced alterations in UI

CS exposure induced a significant (*(*P*<0.01)*) increase in the scores of UI. Pre-treatment with EASF of *M. alba* (25, 50 and 100 mg/kg) significantly decreased the UI in comparison with CS (*P* < 0.01). Administration of diazepam (1 mg/kg) significantly (*P* < 0.01) decreased the UI [Table 5].

**Table 5:** Effect of EASF of *M. alba* on chronic stress-induced ulcerations

*Treatment (mg/kg, p.o.)*	*Number of ulcers*	*Severity of ulcers*	*UI*	*Ulcer incidence (%)*
Control	0	0	0	0
Vehicle	20.0 ± 1.6	36.6	6.6**^a^	100
Diazepam (1)	11.3 ± 1.3	25.1	3.7**^b^	56.6
EASF (25)	7.8 ± 0.7	20.4	2.6*^b^	39.1
EASF (50)	7.5 ± 0.5	20.0	2.5**^b^	37.5
EASF (100)	6.6 ± 0.6	18.8	2.2**^b^	33.3

Values are expressed as mean ± SEM (n = 6),

a Compared with control treated group,

b Compared with chronic stress group

* *P* < 0.05,

** *P* < 0.01 (One-way ANOVA followed by Dunnett's test.)

### Effect of EASF of M. alba on CS-induced alterations in organ weight

Exposure to CS significantly increased (*P* < 0.01) the adrenal gland weight. Pre-treatment with EASF of *M. alba* (25, 50 and 100 mg/kg) significantly (*P* < 0.01) attenuated an increase in the adrenal gland weight. Diazepam (1 mg/kg) also decreased the adrenal gland weight (*P* < 0.01).

A significant decrease was found after CS exposure in spleen weight (*P* < 0.01). Pre-treatment with EASF of *M. alba* (100 mg/kg) significantly (*P* < 0.01) increased the spleen weight. Diazepam was not found to be effective to restore CS-induced decreased spleen weight to a significant extent [Table 6].

**Table 6:** Effect of EASF of *M. alba* on chronic stress-induced changes in the adrenal gland and spleen weights

*Treatment groups (mg/kg, p.o.)*	*Adrenal gland weight (mg/100 g)*	*Spleen weight (mg/100 g)*
Control	17.0 ± 1.2	649.8 ± 10.9
Vehicle	34.3 ± 3.6**^a^	534.8 ± 14.6**^a^
Diazepam (1)	25.3 ± 1.5*^b^	573.2 ± 7.8
EASF (25)	21.4 ± 2.9**^b^	550.3 ± 12.2
EASF (50)	20.1 ± 0.7**^b^	560.4 ± 2.5
EASF (100)	21.1 ± 1.2**^b^	641.8 ± 20.1**^b^

Values are expressed as mean ± SEM (*n* = 6),

a Compared with control treated group,

b Compared with chronic stress group,

* *P*< 0.05,

** *P* < 0.01 (One-way ANOVA followed by Dunnett's test.)

## Discussion

Research on stress in laboratory animals has assured an important role in understanding the biological and behavioral consequences of external and internal stressors that threaten to perturb homeostasis and may induce a number of clinical diseases when the body fails to counter the stress situation. However, it is now widely accepted that chronic inescapable intermittent stress, particularly of an unpredictable pattern, is more likely to induce nervous, endocrine, biochemical and immune changes than acute stress (AS) or CS of a predictable nature.[21]

When stress is applied for a short duration of time, i.e. AS, the physiological changes are self-limiting and adaptive in nature and in this stress, the body sets in motion an array of physiological, biochemical and endocrine responses to counter stress effects.[16] However, if the stress is applied for a prolonged period of time, i.e. CS, the body fails to adapt and results in stress-related illnesses like cognitive dysfunction, behavioral depression, anxiety, hyperglycemia, increase in serum corticosterone level, gastric ulcerations, immunosuppression and increased oxidative stress.[22]

A group of plant-based drugs, the adaptogens, appear to induce a state of non-specific resistance, enabling the organism to counteract and adapt to various stressors. The general aim of adaptogen therapy appears to lie in its ability to reduce stress reactions during the alarm phase of stress response, prevent or at least delay the state of exhaustion and, hence, provide a certain level of protection against long-term stress.[16] Various measures are available to counteract the adverse effects of stress, which include pharmacological and non-pharmacological methods. Use of several antistress agents, particularly the benzodiazepines like diazepam, show significant antistress activity against various models of stress. But, the problems of tolerance and physical dependence exhibited by benzodiazepines, on prolonged use, limit their utility.[21]

The results of the present study revealed that CS induced cognitive dysfunction. Experimental stress was reported to have adverse effects on the memory in rats. The learning and acquisition of learned task was minimally affected, the major effect being disruption of retention of the learned tasks. CS also induces endogenous depression. It has been postulated that cognitive dysfunction and behavioral depression, induced by stress, may be induced by a similar neurochemical mechanism, including depletion of monoamines by sustained stress.[21]

The CS-induced significant increase in blood glucose may be due to close interrelations between stress and the endocrine, autonomic nervous system.[22] The elevated glucose is an energy substrate and helps the organism to combat demand during the stress at its internal and external environment.[20] CS causes a significant increase in the corticosterone level. Activation of the hypothalamo–pituitary–adrenocortical (HPA) axis during stress is a well-known phenomenon and increase in corticosterone in rodents and cortisol in humans have been utilized as markers of stress.[16] Gastrointestinal ulceration appears to be an inevitable consequence of stress, the intensity of ulceration depending on the duration of stress situation.[16] CS leads to prolonged activation of the HPA-axis and results in increased ulcer incidence due to involvement and hyperactivation of the paraventricular nucleus in the hypothalamus.[20] CS-induced adrenal hypertrophy is also a result of the activation of the HPA-axis, which is highly responsive to stress and is one of the principle mechanisms by which an organism mobilizes its defense against stress events.[23] Stress results in the recruitment of lymphocytes to the blood from the spleen and causes squeezing of the spleen, which results in its atrophy and a decrease in the weight.[24]

The pre-treatment with EASF of *M. alba* significantly reversed the memory deficit induced by stress at all doses thus indicating its usefulness in overcoming perturbations of CS on memory. Diazepam did not show any significant improvement in memory. As sustained stress produces depletion of monoamines in the brain, this leads to memory deficit and depression. The improvement in learning task and retention of memory by EASF of *M. alba* may be due to an increase in the levels of depleted monoamines.

Chronic administration of EASF of *M. alba* significantly decreased the duration of immobility. Thus, the extract was effective in relieving endogenous depression. Diazepam also improved stress-induced depression. The antidepressant activity of the extract may be exerted in a similar manner by the increase in the level of depleted monoamines.

Administration of EASF of *M. alba* significantly reversed the elevated glucose level. Diazepam also reduced hyperglycemia. EASF of *M. alba* significantly decreased the adrenal gland hypertrophy and also significantly improved atrophy of the spleen.

Pre-treatment with EASF of *M. alba* significantly reversed the stress-induced UI. The decrease in the UI shows its potential role in attenuating the activation of the HPA-axis during stress. This demonstrates its potential antistress activity. Diazepam also inhibited the ulcerogenic effects of CS.

## Conclusion

The results of the study indicate that EASF of *M. alba* roots possess significant antistress activity, as shown by its mitigating effects on CS-induced neurological, behavioral and biochemical perturbations. Thus, *M. alba* may provide an alternative to conventional therapy for the treatment of stress.
